# Cytotoxicity and cellular uptake of tri-block copolymer nanoparticles with different size and surface characteristics

**DOI:** 10.1186/1743-8977-9-11

**Published:** 2012-04-30

**Authors:** Sourav Bhattacharjee, Dmitry Ershov, Kleanthis Fytianos, Jasper van der Gucht, Gerrit M Alink, Ivonne M C M Rietjens, Antonius T M Marcelis, Han Zuilhof

**Affiliations:** 1 Laboratory of Organic Chemistry, Dreijenplein 8, Wageningen University, 6703 HB, Wageningen, The Netherlands; 2 Division of Toxicology, Tuinlaan 5, Wageningen University, 6703 HE, Wageningen, The Netherlands; 3 Laboratory of Physical Chemistry and Colloid Science, Dreijenplein 6, Wageningen University, 6703 HB, Wageningen, The Netherlands

**Keywords:** Polymer nanoparticles, Cytotoxicity, Mitochondria, Oxidative stress, Intracellular uptake

## Abstract

**Background:**

Polymer nanoparticles (PNP) are becoming increasingly important in nanomedicine and food-based applications. Size and surface characteristics are often considered to be important factors in the cellular interactions of these PNP, although systematic investigations on the role of surface properties on cellular interactions and toxicity of PNP are scarce.

**Results:**

Fluorescent, monodisperse tri-block copolymer nanoparticles with different sizes (45 and 90 nm) and surface charges (positive and negative) were synthesized, characterized and studied for uptake and cytotoxicity in NR8383 and Caco-2 cells. All types of PNP were taken up by the cells. The positive smaller PNP_45_ (45 nm) showed a higher cytotoxicity compared to the positive bigger PNP_90_ (90 nm) particles including reduction in mitochondrial membrane potential (ΔΨ_m_), induction of reactive oxygen species (ROS) production, ATP depletion and TNF-α release. The negative PNP did not show any cytotoxic effect. Reduction in mitochondrial membrane potential (ΔΨ_m_), uncoupling of the electron transfer chain in mitochondria and the resulting ATP depletion, induction of ROS and oxidative stress may all play a role in the possible mode of action for the cytotoxicity of these PNP. The role of receptor-mediated endocytosis in the intracellular uptake of different PNP was studied by confocal laser scanning microscopy (CLSM). Involvement of size and charge in the cellular uptake of PNP by clathrin (for positive PNP), caveolin (for negative PNP) and mannose receptors (for hydroxylated PNP) were found with smaller PNP_45_ showing stronger interactions with the receptors than bigger PNP_90_.

**Conclusions:**

The size and surface characteristics of polymer nanoparticles (PNP; 45 and 90 nm with different surface charges) play a crucial role in cellular uptake. Specific interactions with cell membrane-bound receptors (clathrin, caveolin and mannose) leading to cellular internalization were observed to depend on size and surface properties of the different PNP. These properties of the nanoparticles also dominate their cytotoxicity, which was analyzed for many factors. The effective reduction in the mitochondrial membrane potential (ΔΨ_m_), uncoupling of the electron transfer chain in mitochondria and resulting ATP depletion, induction of ROS and oxidative stress likely all play a role in the mechanisms behind the cytotoxicity of these PNP.

## Background

With the rapid appearance of nanotechnology-based products in the consumer market, human exposure to nanoparticles (NP) is unavoidable [1]. However, a serious lack of knowledge regarding the health and safety issues of these nanotechnology-based products is genuinely felt. A very important question in nanotoxicological research concerns the factors that determine the cytotoxicity of nanomaterials. Obviously, one of the factors is related to size. Due to their small size, NP have a high surface area to mass ratio, which may play a role in the interactions of NP with biomolecules (proteins, cell wall constituents, etc.) and in mechanisms underlying their toxicity when compared to undissolved bulk material. These mechanisms can involve chemical reactions and physical adsorption processes with different biomolecules. Both can ultimately lead to cellular uptake [2] and (cyto)toxic effects [3]. So far, little is also known on the mechanism of cellular uptake and intracellular distribution of different NP inside cells, and how factors like size can influence these.

Currently, many applications are foreseen in fields like NP-based drug delivery and bioimaging [4-6], and for food-based applications [7]. Especially for drug delivery applications, the use of polymer nanoparticles (PNP) is emerging as promising [8]. Recent advancements in polymer science allow synthesis of well-defined polymers (including tri-block copolymers) that can be tailor-made for specific purposes, like drug or food ingredient delivery and biodegradable polymers. Additionally, these tri-block copolymers can be tagged with fluorescent probes to render them fluorescent and thus traceable in biological environments. Thus, PNP derived from the tri-block copolymer can be utilized in the encapsulation of drugs or bioactive food ingredients, and hence can be exciting for drug or food ingredient delivery and sustained release preparations. Although much research is done on the synthesis of biologically valuable PNP, knowledge is lacking on how chemical and physical characteristics like size and charge influence the toxicity and bio-interactions of these PNP.

Therefore, it is essential to investigate how size and charge affect the cytotoxicity as well as other facets of NP-cell interactions, like cellular uptake. In order to interpret the results of such studies, it is essential that the investigated NP are well-characterized and comparable, so that only the size is different, while other factors like composition, surface groups, charge, etc. remain constant. Therefore, in this study particular care was taken that both the polymers and the PNP prepared thereof are well-defined and well-characterized to ensure that differences in biological properties only result from size differences for particles with the same surface groups.

The mechanism of nanomaterial toxicity is not completely clear, and it is possible that more than one mechanism is involved. Literature supports oxidative stress as being an important factor in the mechanism [9-15]. However, it remains to be established if oxidative stress is the mechanism underlying the NP induced cytotoxicity, or a phenomenon accompanying this cytotoxicity. Recently, it was recognized that mitochondria can interact with charged NP, which can then influence the electron transport chain (ETC) [16], although the mechanism underlying this interaction and its exact consequences remain largely unknown. It is possible that due to the damage on the mitochondrial membrane as well as the disruption of the ETC caused by the PNP, the resulting oxidative stress may cause the production of different cytokines (like tumor necrosis factor-α/TNF-α) [17], which in turn is known to be a biomarker of inflammation.

Previously, it was postulated that NP can enter cells by passive diffusion [18] or adhesion [19]. However, this model has proven inadequate in explaining several findings. For example, this model fails to explain why negative NP, which should be repelled by the negative cell membrane, can enter cells in overwhelming amounts [20,21]. Recently, receptor-mediated endocytosis was found to be crucial for the cellular uptake of different NP [2,22,23]. It is possible that cellular uptake of different PNP occurs through different cell membrane-bound receptors, like clathrin and caveolin receptors. These NP-receptor interactions have been related to antigen-antibody coupling reactions [24]. It is hypothesized that surface-functionalized and charge-bearing NP present an “epitope”-like structure, which is recognized and bound to the binding sites of different cell membrane-bound receptors. This initiates a cascade of reactions by which the NP are internalized. Therefore, a detailed investigation on the role of NP-receptor interactions in the cellular uptake of NP is justified with an aim to understand how size and surface charge influences cytotoxicity as well as cellular uptake of different PNP. The cellular in vitro models chosen were rat macrophage NR8383 cells and human colonic adenocarcinoma-derived Caco-2 cells. They represent models for two important targets for NP toxicity upon oral exposure including the innate immune response by phagocytosing cells and human enterocytes.

In this article, a method is described for the synthesis of well-characterized fluorescent PNP of different sizes (45 and 90 nm) and different surface charges from their corresponding tri-block copolymers. The presence of a fluorescent probe in the interior of the PNP makes investigations like bioimaging through confocal laser scanning microscopy (CLSM) possible. The influence of size and charge of the PNP on cytotoxicity as well as on intracellular uptake was studied with a variety of cytotoxicological tools. The influence of cell membrane-bound receptors in the internalization of PNP was investigated with receptor blocking studies and further visualization with CLSM.

## Results

### Synthesis of fluorescent polyethylene glycol_400_-polyhexylene adipate-polyethylene glycol_400_ [PEG_400_-PHA-PEG_400_] (Pol_400_) polymer

**Figure 1:**
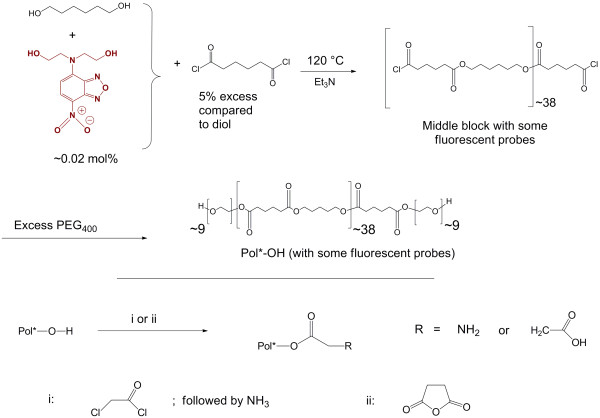
**Overview of the synthesis of PEG**_**400**_**-PHA-PEG**_**400**_**(Pol**_**400**_**) polymer with fluorescent probe and further conversion of the terminal hydroxyl groups to different functional end groups.**

The reaction scheme for the synthesis of Pol_400_ is depicted in Figure 1. The synthesis is similar to the previously reported [4] synthesis of fluorescent Pol_2000_ polymer [polyethylene glycol_2000_-polyhexylene adipate-polyethylene glycol_2000_. From proton nuclear magnetic resonance (^1^ H NMR) (Additional file 1) analysis of the polymer, an estimation of the molecular weight (~9 kDa) was made, which was in agreement with the data obtained from size exclusion chromatography (SEC) (Additional file 2). From the infrared (IR) spectrum of the polymer (Additional file 3) the carbonyl (C = O) stretch from the polyester middle block could easily be seen at 1737 cm^-1^. From these combined data it is inferred that the middle block has about 35-40 repeating units. From SEC, a polydispersity index (PDI = *M*_*w*_*/M*_*n*_ where *M*_*w*_ = molecular weight; *M*_*n*_ = relative molecular weight) of 1.47 was obtained for Pol_400_ (Table 1). Based on the average chain length of the polymer molecules as well as the initial amount of probe, it is estimated that roughly 1 % of the polymer molecules contained a fluorescent probe, embedded in the hydrophobic middle block.

**Table 1:** **Data of Pol**_**400**_**with its end group conversion rates**

***Polymer***	***Molecularweight (kDa)***	***Meltingpoint (***^***o***^*C)*	***PDI***	***Converted to***	***Conversion (%)***
***Pol***_***400***_	^***1***^***H NMR*** 9.0	43	1.47	Pol_400_-NH_2_	> 90
	***SEC*** 9.0			Pol_400_-COOH	> 90

### Conversion of terminal hydroxyls to differently charged end groups

The terminal hydroxyl (-OH) groups of Pol_400_ were converted into an amine by first reacting the polymer with chloroacetyl chloride and subsequently adding concentrated ammonia, which yielded Pol_400_-NH_2_ . Reacting the polymers with succinic anhydride yielded Pol_400_-COOH. In contact with water at neutral pH, this means that the terminal groups will be either positively charged (-NH_3_^+^) or, negatively charged (COO^–^) due to the pK_a_ values of these groups. The degree of conversion of the terminal hydroxyl groups was assessed by reaction with trichloroacetyl isocyanate (TAIC) [25]. Reaction of TAIC with terminal hydroxyl groups gives a characteristic peak in the ^1^ H NMR spectrum at δ = 4.43 ppm. Upon converting these -OH groups to amino or carboxylate moieties, this reaction with TAIC is not possible anymore, and this peak is thus smaller or even absent, depending on the conversion to -NH_3_^+^ or -COO^–^. This analysis was used to quantify this conversion (Table 1 and Additional file 4), which turned out to be almost quantitative for all polymers. The data on the characterization of Pol_400_, including the conversions of the end hydroxyl groups to different functional groups (-NH_2_ and -COOH) are given in Table 1. The melting points of all polymers were determined by differential scanning calorimetry (DSC) and were found to be ~43°C for all polymers. This indicates that the melting point is only determined by the middle block, which has the same length in all synthesized polymers.

### Synthesis and characterization of different PNP

**Figure 2:**
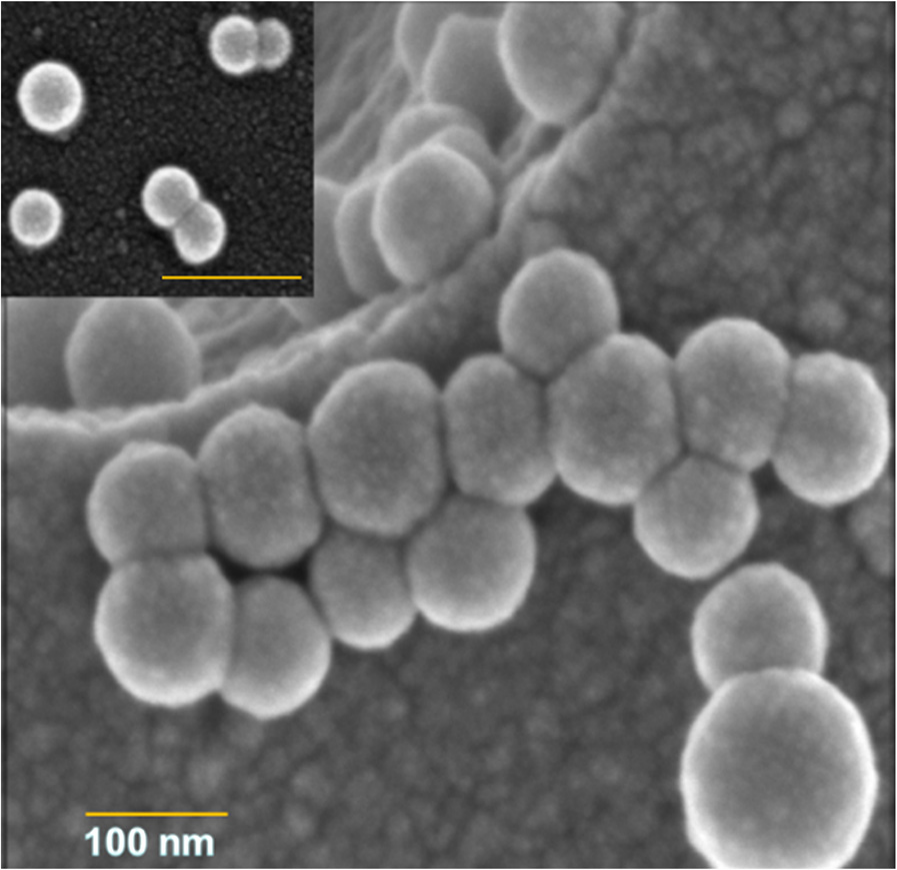
**SEM image of monodisperse PNP**_**90**_**(90 ± 5 nm) and PNP**_**45**_**(45 ± 5 nm) (as insert).** Scale bars are 100 nm.

The different PNP_90_ were prepared by nanoprecipitation, where a solution of Pol_400_ polymer in tetrahydrofuran (THF) was injected into vigorously stirred water. Similarly, different PNP_45_ were obtained from Pol_2000_[4]. This resulted in a stable clear aqueous dispersion of PNP_90_-X or, PNP_45_-X (X = NH_2_, OH and COOH). The size of PNP_90_ was 90 ± 5 nm as determined by scanning electron microscopy (SEM) (Figure 2) and supported by dynamic light scattering (DLS). Both DLS and SEM data showed particles of comparable size and also reveal that their structural integrity is maintained, even upon drying. The SEM pictures of PNP_90_-NH_2_, PNP_90_-OH and PNP_90_-COOH are provided as Additional file 5. The average ζ-potential of these PNP_90_ in aqueous dispersions (0.1 μg/ml) were found to be +22 mV for PNP_90_-NH_2_, -4 mV for PNP_90_-OH and -19 mV for PNP_90_-COOH. The hydrodynamic sizes of these PNP were also determined by DLS in F12-K and DMEM medium (0.1 μg/ml) that contained fetal calf serum (FCS). Upon addition of these PNP_90_ into cell culture mediums, the sizes increased mainly due to surface adsorption of proteins although the polydispersity did not increase considerably. The DLS data of different PNP_90_ are given in Table 2.

**Table 2:** **DLS data of PNP**_**90**_

***Type***	***Size in water (nm)***	***Size in F12-K (nm) (after 1 h)***	***Size in DMEM (nm) (after 1 h)***
*PNP*_*90*_*-NH*_*2*_	*90 ± 5*	*145 ± 5*	*140 ± 5*
*PNP*_*90*_*-OH*	*90 ± 5*	*115 ± 5*	*120 ± 5*
*PNP*_*90*_*-COOH*	*90 ± 5*	*120 ± 5*	*130 ± 5*

### Evaluation of the cytotoxicity of nanoparticles

#### A. MTT assay

**Figure 3:**
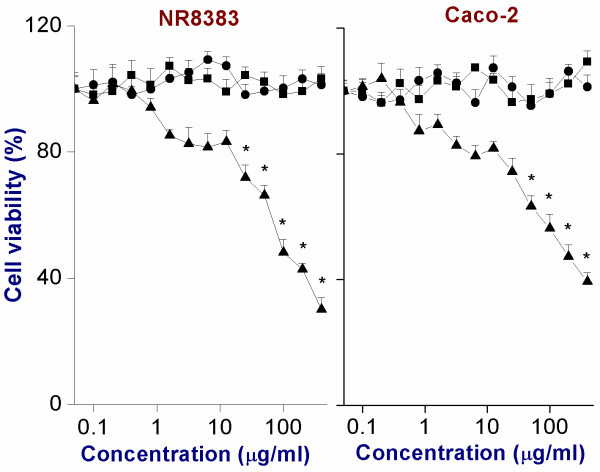
**Cytotoxicity of PNP**_**90**_**-NH**_**2**_**(▲), PNP**_**90**_**-OH (■) and PNP**_**90**_**-COOH (●) on NR8383 and Caco-2 cells as measured by the MTT assay after 24 h exposure.** The * sign signifies *p* < 0.05 compared to negative control (0 μg/ml).

The cell viability of the different PNP_90_ was determined in two different cell lines, i.e. NR8383 and Caco-2 cells. The PNP_90_ were studied in the concentration range of 0-400 μg/ml after 24 h exposure and the results were compared with the data obtained previously for PNP_45_[4]. These data are shown in Figure 3. Positively charged PNP_90_-NH_2_ were cytotoxic within the tested concentration range, whereas the negatively charged ones were not. The PNP_45_-NH_2_ were more cytotoxic than PNP_90_-NH_2_ as can be seen from the EC_50_ values (Table 3). Upon exposure to the positive control Triton-X (0.1 %) NR8383 and Caco-2 cells both showed a cell viability of 1 % compared to their viability upon exposure to the negative control (0 μg/ml).

**Table 3:** **EC**_**50**_**values (μg/ml) obtained from different assays after 24 h exposure of NR8383 and Caco-2 cells to positively charged PNP**_**45**_**[**[4]**] and PNP**_**90**_

***Assay***	***Parameter***	***Reference figure***	***Cell line***	***PNP***_***90***_***-NH***_***2***_***(90 ± 5 nm)***	***PNP***_***45***_***-NH***_***2***_***(45 ± 5 nm)***
MTT	Cell viability	3	NR8383	55	31
			Caco-2	68	54
PI	Phagocytosis	4	NR8383	80	64
DCFH-DA	Intracellular ROS production	5	NR8383	23	13
			Caco-2	33	21
Mitochondrial membrane potential (ΔΨ_m_)	Mitochondrial membrane potential	6	NR8383	5	2
			Caco-2	6	3
ATP	Cellular ATP content	7	NR8383	26	14
			Caco-2	62	36
TNF-α	TNF-α release	8	NR8383	37	25
			Caco-2	63	32

### B. Phagocytic Index (PI) measurement

**Figure 4:**
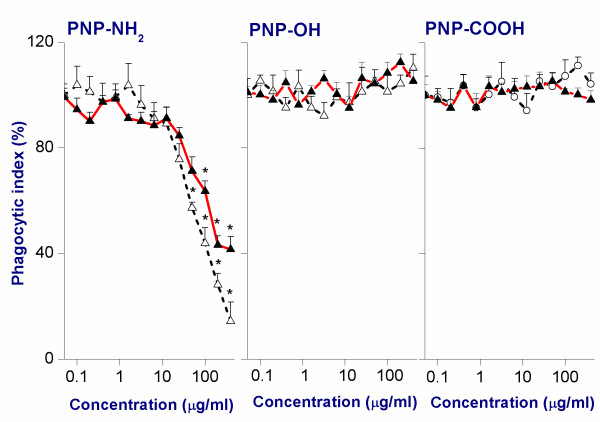
**Phagocytic Index (PI) in NR8383 cells after 24 h exposure to different PNP**_**45**_**(Δ) and PNP**_**90**_**(▲).** The * sign signifies *p <* 0.05 compared to negative control (0 μg/ml).

The PI for macrophage NR8383 cells was determined by measuring the capability of the cells to phagocytose 1 μm fluorescent latex beads (see Figure 4). Like in the MTT assay, the positive PNP showed signs of cytotoxicity by effecting a decrease of the PI upon increasing the concentration of PNP, whereas the negative PNP did not show any effect at all in the tested concentration range. The PNP_45_-NH_2_ was relatively more cytotoxic than PNP_90_-NH_2_, as can be derived from the EC_50_ values (Table 3). The NR8383 cells exposed to the positive control (100 μM CuSO_4_) showed ~1 % PI compared to the NR8383 cells exposed to the negative control (0 μg/ml).

### Assessment of intracellular reactive oxygen species (ROS) production by DCFH-DA assay

**Figure 5:**
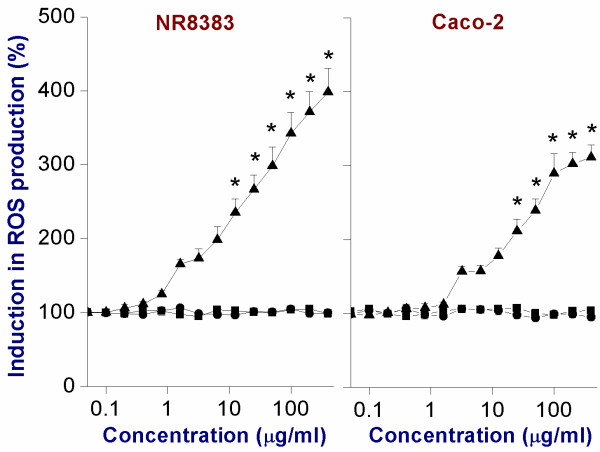
**Intracellular ROS induction in NR8383 and Caco-2 cells after 24 h exposure to PNP**_**90**_**-NH**_**2**_**(▲), PNP**_**90**_**-OH (■) and PNP**_**90**_**-COOH (●).** The * sign signifies *p <* 0.05 compared to negative control (0 μg/ml).

PNP with chemically reactive surfaces can interact with biological molecules resulting in production of radicals including reactive oxygen species (ROS), which in turn can cause toxicity. Production of ROS can be tested with the DCFH-DA assay, which measures the intracellular production of ROS. Positive PNP_90_-NH_2_ were able to induce intracellular ROS production in both NR8383 and Caco-2 cells, whereas negative PNP (both PNP_90_-OH and PNP_90_-COOH) did not (Figure 5). Furthermore, in both NR8383 and Caco-2 cells, the induction of intracellular ROS production with PNP_45_-NH_2_ was stronger than with PNP_90_-NH_2_ (see Table 3 for EC_50_ values). These results match the results of the MTT assay and indicate a possible relation between oxidative stress and cell viability. Exposure of the NR8383 and Caco-2 cells to the positive control (10 mM H_2_O_2_) caused respectively ~1000 % and ~900 % induction of ROS production compared to the level of ROS production (100 %) in cells exposed to the negative control (0 μg/ml)

### Assessment of mitochondrial membrane potential (ΔΨ_m_)

**Figure 6:**
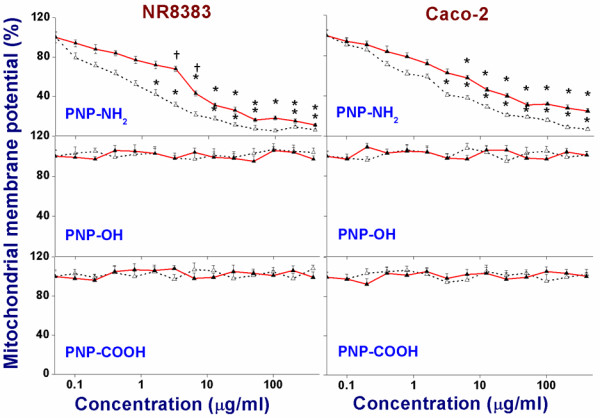
**Mitochondrial membrane potential (ΔΨ**_**m**_**) of NR8383 and Caco-2 cells after 24 h exposure to different PNP**_**45**_**(Δ) and PNP**_**90**_**(▲) as % of negative control (0 μg/l).** The * sign signifies *p <* 0.05 compared to negative control (0 μg/ml). The “†” sign signifies *p <* 0.05 when compared between the PNP_45_ and PNP_90_.

Figure 6 shows the results from assessment of the change in mitochondrial membrane potential (ΔΨ_m_) in NR8383 and Caco-2 cells exposed to increasing concentrations of PNP_45_ or PNP_90_ with different charges. Only the cationic PNP (-NH_2_ terminated) of both sizes, showed signs of decreasing the ΔΨ_m_ for both the NR8383 and Caco-2 cells, whereas the anionic ones (-OH and -COOH terminated) did not show any effect. The EC_50_ values are given in Table 3. The smaller cationic PNP_45_ were more effective, reflected by lower EC_50_ values, compared to the bigger PNP_90_. This reduction in mitochondrial membrane potential (ΔΨ_m_) may affect ATP generation in the cells, thereby contributing to the mode of action for the cellular toxicity. Exposure of the cells to the positive control (100 μM ionomycin) caused a decrease of ΔΨ_m_ to < 2 % of the ΔΨ_m_ detected in cells exposed to the negative control (0 μg/ml).

### Assessment of intracellular ATP content

**Figure 7:**
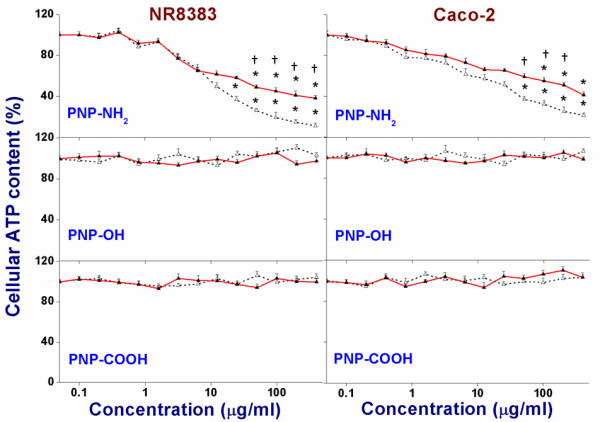
**Effect on cellular ATP content in NR8383 and Caco-2 after 24 h exposure to different PNP**_**45**_**(Δ) and PNP**_**90**_**(▲) as % of negative control (0 μg/l).** The * sign signifies *p <* 0.05 compared to negative control (0 μg/ml). The “†” sign signifies *p <* 0.05 when compared between the PNP_45_ and PNP_90_.

As a consequence of interaction of charged NP with mitochondria, disruption of the electron transport chain, reflected by a reduction of the mitochondrial membrane potential (ΔΨ_m_), can occur. This may result in ROS production and in depletion of the cellular ATP content; the latter being an (additional) possible mechanism of cytotoxicity. Therefore, the intracellular ATP content of cells after exposure to PNP was determined. The intracellular ATP content after 24 h exposure to different PNP_90_ and PNP_45_ is shown in Figure 7 and the EC_50_ values are given in Table 3. Both NR8383 and Caco-2 cells showed a gradual dose-dependent decrease in intracellular ATP content only upon exposure to the positive PNP. This ATP depletion was more profound for PNP_45_ than for PNP_90_. Exposure of the cells to the positive control (75 mM 2,4-dinitrophenol/DNP) caused a decrease of intracellular ATP to < 2 % of the levels in cells exposed to the negative control (0 μg/ml).

### Assessment of TNF-α production

**Figure 8:**
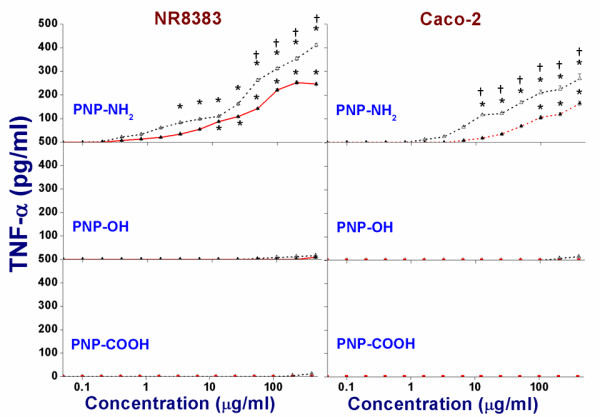
**Comparison of TNF-α release induced by PNP**_**45**_**(Δ) and PNP**_**90**_**(▲) in NR8383 and Caco-2 cells after 24 h exposure.** The * sign signifies *p <* 0.05 compared to negative control (0 μg/ml). The “†” sign signifies *p <* 0.05 when compared between the PNP_45_ and PNP_90_.

TNF-α is a major biomarker cytokine for pro-inflammatory response. It can stimulate an acute phase reaction as well as apoptosis in living tissue [26]. Hence, a surge in the production of intracellular TNF-α indicates inflammation, which can also be a factor for the toxicity caused by PNP. The TNF-α production in NR8383 cells was measured for both sizes of PNP (45 and 90 nm) after 24 h exposure (see Figure 8). Only positive PNP of both sizes showed significant induction of the TNF-α production. As found in the other cytotoxicity experiments, the results again indicate that the smaller positive PNP_45_ were more toxic than positive PNP_90_. The corresponding EC_50_ values are listed in Table 3. Exposure of the cells to the positive control (lipopolysaccharide/LPS) caused an increase of TNF-α to > 900 pg/ml in both the NR8383 and Caco-2 cells.

### Intracellular uptake of PNP_90_

**Figure 9:**
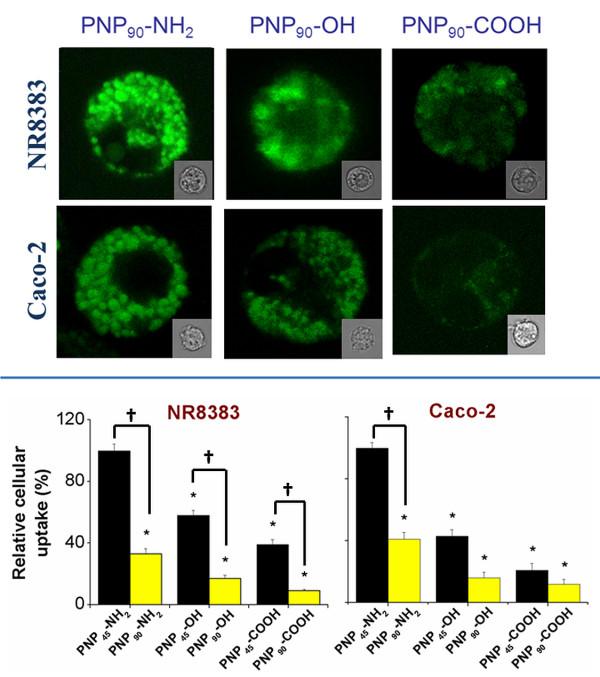
**(Upper layer)CLSM pictures of NR8383 and Caco-2 cells after 24 h exposure to a non-toxic concentration of 1 μg/ml of different PNP**_**90**_** (λ**_**ex**_** = 488 nm and λ**_**em**_** = 543 nm) with phase contrast figures given as inserts.(Lower layer)** Relative cellular uptake of different PNP_45_[4] and PNP_90_ in NR8383 and Caco-2 cells after 24 h exposure with the PNP_45_-NH_2_ (1 μg/ml) values taken as 100 % (n = 5). The * sign signifies *p <* 0.05 when compared to the data for PNP_45_-NH_2_. The “†” sign signifies *p <* 0.05 when compared between the PNP_45_ and PNP_90_.

The intracellular uptake of different PNP_90_ could be monitored by CLSM (*λ*_*ex*_ = 488 nm and *λ*_*em*_ *=* 543 nm) as these PNP carried a fluorescent probe. All the CLSM experiments were performed at a concentration of 1 μg/ml of PNP that was non-toxic, as determined by the MTT assay. In Figure 9 (Upper layer) representative CLSM images are given that show the relative intracellular uptake of these three different PNP. From the quantitative uptake results in Figure 9 (Lower layer) it follows that all PNP_90_ were taken up intracellularly, with PNP_90_-NH_2_ showing the highest and PNP_90_-COOH the lowest cellular uptake. Interestingly, a surface charge-dependent intracellular distribution of these PNP is observed. Only the positive PNP_90_-NH_2_ showed stronger interactions with the cellular periphery, whereas the PNP_90_-OH and PNP_90_-COOH showed a more diffuse uptake in the cytoplasm. Upon exposure of the cells to equal concentrations of the different PNP and integration of the fluorescence over several cells, a comparison between the uptake of the PNP_90_ and PNP_45_ particles with different surface charges could be made (see Figure 9 lower layer). The CLSM data were normalized for the uptake of PNP_45_-NH_2_. It is seen from this figure that the intracellular uptake of PNP_45_-NH_2_ was about three times (for NR8383) and two times (for Caco-2) higher than of PNP_90_-NH_2_. Similar size-dependent effects were found for both -OH and -COOH terminated PNP.

### Effect of size and surface charge on endocytosis-based cellular uptake of PNP

**Figure 10:**
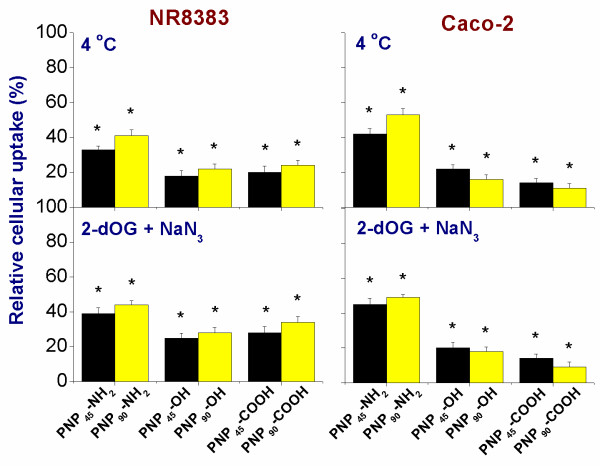
**Comparison of uptake of different PNP**_**45**_**[**[4]**] and PNP**_**90**_**in NR8383 and Caco-2 cells (1 μg/ml) as % of unperturbed uptake upon 24 h exposure after blocking the endocytotic uptake (n = 5) at 4 ºC or by a mixture of 2-deoxyglucose and sodium azide.** The * sign signifies *p <* 0.05 compared to unperturbed uptake.

The role of endocytosis in the cellular uptake of different PNP_90_ was tested by inhibiting endocytotic pathways either by lowering the experimental temperature to 4 ^o^C or by exposing the cells to a mixture of 2-deoxyglucose (2-dOG) and sodium azide (NaN_3_) (see Figure 10). The blocking of endocytosis by either procedure drastically reduced the cellular uptake of PNP_90_, irrespective of the surface charge. However, the decrease was much stronger for PNP_90_-OH and PNP_90_-COOH (>80 %) than for PNP_90_-NH_2_ (~50 %). It was observed that the blockade of endocytosis had a stronger effect on the uptake of PNP_90_-OH and PNP_90_-COOH compared to the PNP_90_-NH_2_. To confirm that the PNP were inside the cells and not bound to the cell membrane, *z*-stack imaging was done. Such a *z*-stack imaging figure is provided as Additional file 6, and shows that PNP were inside the cells apart from being attached to the surface.

### Effect of size and surface charge on clathrin and caveolin mediated endocytosis

**Figure 11:**
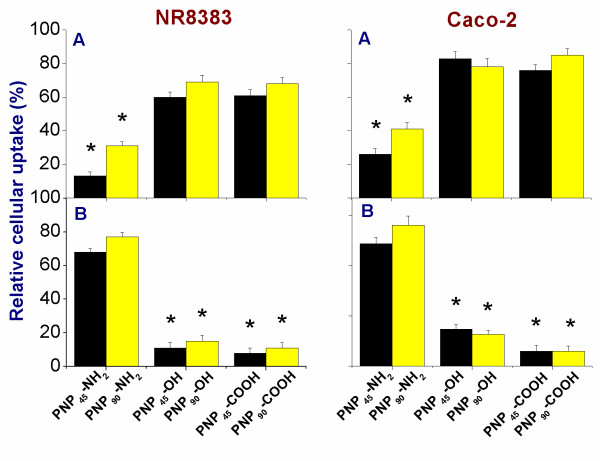
**Comparison of uptake of different PNP**_**45**_**[**[4]**] and PNP**_**90**_**in NR8383 and Caco-2 cells (1 μg/ml) upon 24 h exposure after blocking the clathrin (A) and caveolin (B) receptors (n = 5).** The * sign signifies *p <* 0.05 when compared to unperturbed uptake.

The size and charge-dependent involvement of clathrin and caveolin receptors in endocytosis of different PNP was tested by selectively blocking the clathrin and caveolin receptors and the results are shown in Figure 11. The clathrin receptors were inhibited by exposing the cells to a hypertonic 450 mM sucrose solution, as this causes polymerization and subsequent inactivation of clathrin receptors [27]. From the results (Figure 11A), it is clear that the positive PNP-NH_2_ of both sizes showed a considerably reduced uptake upon blocking the clathrin receptors (effect: PNP_45_ > PNP_90_). In contrast, the uptake for both hydroxylated and acid terminated PNP was only affected to a milder extent (reduction to ~65 % and ~75 % of the original values for PNP-OH and PNP-COOH of both sizes, respectively). An opposite effect was seen when the caveolin receptors were blocked by exposing the cells to MβCD [16] as seen from Figure 11B. A profound decrease in the cellular uptake of both the negative PNP could be seen after blocking the caveolin receptors while uptake of PNP-NH_2_ was only affected slightly. Here also a stronger decrease in uptake was found for the smaller PNP_45_ than for the larger PNP_90_.

### Effect of size and surface charge on mannose receptors

**Figure 12:**
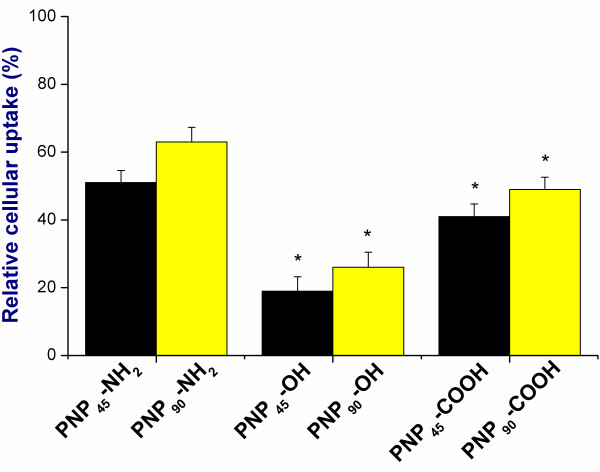
**Comparison of the uptake of different PNP**_**45**_**and PNP**_**90**_**in NR8383 cells (1 μg/ml) upon 24 h exposure after blocking the mannose receptors (n = 5).** The * sign signifies *p <* 0.05 compared to unperturbed uptake.

The role of mannose receptors in the intracellular uptake of different PNP was investigated by inhibiting the mannose receptors by exposing the cells to a high concentration of α-mannan (see Figure 12). It can be seen that inhibition of the mannose receptors decreased the intracellular uptake for all PNP, but the largest effects were seen for the negative PNP (PNP-OH > PNP-COOH), especially for the smaller ones (PNP_45_ > PNP_90_).

### Statistical analysis

Data were analyzed with Origin Pro (version 8.0) graphing software. For statistical analysis a student’s *t*-test was performed, and data with *p* < 0.05 (compared to negative control) were marked with an asterisk (*) sign. Each data point represents the average from three independent experiments (n = 3) (for CLSM studies n = 5) and is presented as the arithmetic mean ± standard error of mean. While comparing the effect of size, the results for PNP_45_ and PNP_90_ were also mutually compared and only the statistically significantly different (*p* < 0.05) data between the two were marked by “†” symbol.

## Discussion

Due to the nature of the tri-block copolymers synthesized and used in the present study, which have a hydrophobic middle block and hydrophilic terminal blocks, the PNP that were formed out of these polymers in water had a hydrophobic core and a hydrophilic corona. The size of the PNP depends largely on the size of the polymers and the ratio between their hydrophobic middle and hydrophilic terminal blocks [28]. The hydrophobic middle blocks (the polyhexylene adipate polyester) avoid contact with water, whereas the hydrophilic (PEG) blocks try to remain in contact with the water. This means that for the polymers with the larger hydrophilic blocks the aggregate surface becomes crowded with PEG groups faster and the particles stop growing sooner than for the polymers with the smaller terminal groups. The polymers with the smaller PEG groups grow into larger particles, because in that case the surface does not get so easily crowded and more polymer molecules will add to the forming nanoparticle. The MTT assay data suggest that the positive PNP_45_ were more cytotoxic than the positive PNP_90_. Both the smaller and larger negative PNP did not induce any significant cytotoxic response at concentrations up to 12.5 μg/ml. Although some studies have been performed on the size-dependent cytotoxicity of NP, such studies on PNP are rare. In the present study the PNP_45_ and PNP_90_ that were used have very similar properties in surface charge density. To the best of our knowledge, this is the first report in which the effect of size on cytotoxicity of PNP is systematically investigated, while keeping the surface characteristics and PNP composition unchanged.

For inorganic NP more is known on the effect of size on cytotoxicity. Recently, it was reported that smaller gold nanoparticles (1.4 nm) were much more cytotoxic than bigger (15 nm) ones [29]. In a similar experiment, silver NP of three different sizes (15, 30 and 55 nm) were tested on a rat alveolar macrophage cell line, which is comparable to the NR8383 cell line used in this study. The cytotoxicity of the smallest (15 nm) particles was highest and of the biggest ones (55 nm) the lowest [30]. The cytotoxicity of a wide size-range of silica NP (30, 48, 118 and 535 nm) was tested on a mouse keratinocyte HEL-30 cell line. A clear size-dependent cytotoxic pattern was reported. The smaller (30 and 48 nm) silica NP showed a much higher toxicity than the bigger (118, 535 nm) ones [31]. For copper NP similar results were reported [32]. Some other reports also indicated an inverse relationship between size and toxicity of different NP including PNP [13,33]. Auffan et al. [34] hypothesized that inorganic NP smaller than 30 nm are chemically very unstable due to the presence of many high energy surface states which makes them extremely reactive, which again results in an enhanced cytotoxicity. However, many of the surface properties of inorganic NP are significantly different from the surface properties of organic PNP. Therefore, although our results match the findings for inorganic NP, a true comparison is difficult.

It should be noted that in all the cellular experiments reported in this article, cell culture media (DMEM and F12-K) contained FCS rich in proteins (like albumin). It has been observed before that the presence of FCS can cause an increase in the sizes of these PNP by surface adsorption of proteins, although the PNP, in this case, still remained highly monodisperse [4]. The presence of serum, by virtue of being high in protein content, has been reported to influence the toxicity and cellular uptake of NP [3]. This protein adsorption can be of influence on cellular uptake and toxicity, but it is expected that such protein effects will also occur in the gastrointestinal tract upon oral exposure to PNP. Thus, testing in the presence of serum better reflects the physiological conditions for PNP that may be developed with food-based applications in mind.

Two series of PNP were investigated that differ in size and within each series PNP with different surface charges (-NH_2_, -OH and -COOH) were investigated. A distinct size-dependence was observed. For instance, although both amine-terminated PNP were toxic, the smaller PNP_45_ were more cytotoxic than the larger PNP_90_. Hence a size-dependent effect for PNP with comparable surface charge and surface functionalization was observed. In equivalent masses, the smaller PNP_45_ (45 nm) presented two times more surface area compared to the bigger PNP_90_ (90 nm). Upon expressing the toxicity data based on surface area, it was found that the toxicity increased with an increase in PNP surface area.

Production of intracellular ROS after exposure to different NP has been amply reported [35-39]. It is thought that these ROS push the cellular physiology to the limits by inducing oxidative stress. Our data, obtained from the DCFH-DA assays, show that only positive PNP induced intracellular ROS production with the smaller PNP showing a higher effect. These findings also match the pattern of cytotoxicity (MTT assay) for these PNP. In literature, systematic studies on the effect of size on the intracellular ROS production are rare. Jiang et al. [40] investigated the effect of size on intracellular ROS production by testing a wide range of titanium dioxide NP (4-195 nm) and reported the highest ROS induction for NP of 30 nm size. In a separate study, silver NP of 4, 20 and 70 nm were tested on macrophage U937 cells [41]. It was found that the 20 nm silver NP were the most capable of producing oxidative stress. A similar study by Choi et al. [42] showed that within a series of different sizes of silver NP tested, the smallest (5 nm) NP were the most capable of inhibiting the growth of nitrifying bacteria through production of ROS. Landsiedel et al. [43] also mentioned an inverse relationship between the size of NP and their induction of intracellular ROS in their comprehensive review on different metal oxide NP (like CeO_2_, TiO_2_, SiO_2_, ZrO_2_).

The decrease of the mitochondrial membrane potential (ΔΨ_m_) by cationic PNP is an important finding. It shows that cationic PNP can indeed interact with intracellular mitochondria and compromise their integrity. The decrease in ΔΨ_m_ after exposure to cationic PNP can have further consequences. This compromised state of the mitochondrial membrane can increase its permeability which may result in leaching of the mitochondrial calcium to the cytoplasm causing a cellular overload of calcium, release of cytochrome c and subsequently trigger apoptosis [44]. Similarly, a compromised mitochondrial membrane also can hamper the normal electron transport chain. This can result in decreased ATP production. The finding of ATP depletion of cells upon exposure to cationic PNP matches well with the observed effect on the mitochondrial membrane potential (ΔΨ_m_). Previously, Bhattacharjee et al. [14] reported that positive silicon NP (1.6 ± 0.2 nm) were able to induce ROS production in isolated mitochondrial fractions from rat liver tissue. Similarly, Xia et al. [45] observed that cationic polystyrene nano-beads can interact with and subsequently harm intracellular mitochondria. Due to the continuous involvement of mitochondria in the respiratory cycle by virtue of the electron transport chain (ETC) processes occurring on the outer membrane, it was suggested that interaction of positive NP with mitochondrial membranes can disturb the mitochondrial membrane potential. This was shown in the present study to occur upon exposure of the cells to positive PNP. As a result, positive PNP might uncouple the cascade of reactions in the ETC and thus not only hamper ATP production but also increase the intracellular ROS production [14]. A recent study reported that intracellular ATP depletion occurred upon exposure of human endothelial EAhy926 cells to differently sized polystyrene NP [46]. The data obtained in the present study are in line with the literature and this could shed some light on the poorly understood mechanism of intracellular ROS production induced by NP. In our opinion, reduction of the mitochondrial membrane potential (ΔΨ_m_) followed by intracellular ATP depletion, as observed after exposure to cationic PNP, may also be a mechanism of cytotoxicity, related to or independent of intracellular ROS production and warrants further investigation.

It has been reported that several NP can induce production of inflammatory cytokines like TNF-α in different cell lines, including a primary rat brain microvessel cell line and human alveolar epithelial A549 cells [26,47,48]. However, a comparative study on the effect of NP size on TNF-α induction is rare, especially for PNP. Recently, size-dependent TNF-α induction was reported when titanium dioxide NP (5 and 200 nm) were intra-tracheally instilled in rats [49]. It was observed that 5 nm particles were much more effective in inducing TNF-α than 200 nm ones. A similar type of inverse relationship between size of NP and TNF-α production was reported by Hanley et al. [50] for silver oxide NP. Our data on TNF-α production match these reported literature data and point towards an inverse relationship between size of PNP and TNF-α production.

Our data on the cellular uptake show that smaller PNP_45_ (45 nm) were taken up in appreciably larger amounts (as determined by CLSM) than the bigger PNP_90_ (90 nm), irrespective of surface charge. Win et al. [51] reported a similar type of inverse relationship between size of PNP and cellular uptake in Caco-2 cells. Recently, fluorescent and carboxyl-terminated polystyrene NP of 20 and 200 nm sizes were tested on both rat and human primary hepatocyte cells [52] and it was found that the smaller 20 nm polystyrene NP showed a higher intracellular uptake than the bigger ones. Many other groups reported a higher intracellular uptake for smaller NP [51,53-55]. Zhang et al. [56] performed a molecular modeling and thermodynamics study to understand this size dependence. From calculations and thermodynamics they predicted that NP of ~22 nm radius (i.e. ~44 nm in size) are more easily internalized by cells. Similarly, other computational models [57-59] also predicted an energetically favorable receptor-mediated intracellular uptake for NP of 30-50 nm sizes. These authors were also able to predict an upper threshold radius of ~60 nm (i.e. ~120 nm size), where receptor-based endocytosis will not be favorable anymore. These findings fit quite well with our data.

The inhibition of endocytosis (by performing the experiments at 4°C or exposure to a mixture of 2-deoxyglucose and sodium azide) had a stronger effect on the uptake of PNP_45_ than on the uptake of PNP_90_. An explanation may be that smaller PNP enjoy a higher degree of binding with cell membrane receptors. Hence, inhibition of receptor-based endocytosis affects the cellular uptake of smaller particles more. An optimal size of 50 nm was proposed for uptake as well as saturation kinetics of NP uptake by Chithrani et al. [60], who investigated the uptake of gold NP of three different sizes (14, 50 and 74 nm) in HeLa cells [2]. Recently, Jiang et al. [61] also reported that receptor-based endocytosis was highest for 40-50 nm gold and silver NP tested on herceptic receptor ErbB2 expressed on macrophage cells. This preference for 40-50 nm NP also matches our data.

Like in the general receptor inhibition studies, our data show that selective blocking of the clathrin, caveolin or, mannose receptors had in all cases a stronger effect on the uptake of smaller PNP_45_ particles than that of the larger PNP_90_ particles. These results are independent of surface charge of the PNP, although the preference of clathrin receptors for positive PNP and caveolin receptors negative PNP is evident from the results. However, it should also be noted that blockers lack absolute specificity and also that other endocytotic pathways for cellular PNP uptake remain available after blocking one receptor type. Furthermore, because different in vitro cellular systems have different physiologies and have different levels of expression of clathrin or caveolin receptors, results from different studies cannot always be easily compared [62]. Whether combined inhibition of the clathrin, caveolin and mannose receptors would completely abolish the cellular uptake of the PNP, or that residual uptake would remain because also other uptake mechanisms are of importance, remains to be investigated. There is only a limited amount of systematically performed size-dependent analyses on interactions of PNP with endocytosis receptors. Rejman et al. [63] performed a very extensive study on cellular uptake mechanisms of latex particles in murine melanoma B16-F10 cells and found a preference for clathrin receptors by smaller and caveolin receptors by bigger PNP. More recently, it was reported that carboxyl-terminated polystyrene NP of 43 nm size got internalized by the cells through a clathrin-dependent pathway [54]. Oh et al. [64] also found that uptake of smaller metal hydroxide nanoparticles (50, 109, 200 nm), when tested on a human osteosarcoma (MNNG/HOS) cell line, showed a stronger clathrin receptor dependence of cellular uptake than the bigger ones (375 nm). So, although the effects are clearly different for the differently charged PNP and the different receptors, our results seem to be in line with the findings of the majority of these reports that smaller (or medium-sized) NP have stronger interactions with endocytosis receptors than larger NP.

Mannose receptors are a unique group of receptors that are often expressed on macrophage cell surfaces and recognize and endocytose a wide variety of carbohydrates. Though it is not clear yet how these receptors recognize such a huge variety of molecules, the orientation of the carbohydrate molecule is important. It is also known that hexoses with equatorially placed hydroxyl groups have a strong binding affinity towards these receptors [65].

Although our PNP do not contain carbohydrate groups, especially those that contain –OH groups on the surface have some chemical resemblance with carbohydrates with -OH groups. The results of the inhibition studies match with this, since the maximum inhibition of intracellular uptake was observed for both hydroxyl- or acid-terminated PNP. This once again points towards a complex range of interactions between PNP and cell membranes and their receptors leading to particle internalization. Although we are the first to actually show that inhibition of mannose receptors inhibits cellular uptake of PNP, there have been a few reports already where these receptors have been targeted for facilitated drug delivery. Park et al. [66] used mannosylated polyethyleneimine coupled to silica NP to increase the transfection efficiency in macrophage cells by targeting the mannose receptors. Similar strategies have been employed by other groups to increase the delivery efficacy in biological systems [67-69]. Our results on mannose receptors are in line with data available in literature that point towards strong NP-receptor interactions. These results can be further developed for more sophisticated applications like drug delivery or food-based delivery of functional ingredients.

## Conclusions

Well-characterized, monodisperse and fluorescent PNP of different sizes (45 and 90 nm) and surface properties were synthesized. The PNP exhibited an inverse relationship between size and cytotoxicity as well as between size and cellular uptake. A size-dependent induction of intracellular ROS production identifies oxidative stress as a possible mechanism of cytotoxicity with subsequent release of inflammatory cytokines representing another mechanism for NP induced adverse effects. Reduction in the mitochondrial membrane potential, uncoupling of the electron transfer chain in mitochondria and resulting ATP depletion, induction of ROS and oxidative stress likely all play a role in the mode of action for the cytotoxicity of these PNP.

Although at the concentrations tested, only positive PNP show cytotoxic effects, all PNP were taken up by the cells. An involvement of clathrin, caveolin and mannose receptors could be seen in cell internalization of PNP with their relative importance depending on the surface properties of the PNP. Overall the results presented provide insight in size and surface charge-specific cellular uptake and cytotoxicity of PNP and possible modes of action underlying these effects. Typically, larger PNP and negatively charged PNP are less toxic in our tests than smaller and positively charged PNP. Translation of these results to recommendations for their preferential use in development of safer NP requires validation of their lower toxicity in vivo studies.

## Methods

### Synthesis of the fluorescent tri-block copolymers

The fluorescent probe, 4-(diethanolamino)-7-nitro-benzo-[1,2,5]oxadiazole, was synthesized and characterized as reported in literature [70]. For the synthesis of Pol_400_, a dry 100 ml three-necked round-bottom flask containing properly grinded and dried 1,6-hexanediol (2.0 g; 17 mmol) and 0.1 mg of fluorescent probe (~4 × 10^–4^ mmol) was fitted with a reflux condenser and flushed with dry nitrogen for 30 min. Subsequently, the flask was heated on an oil bath at 120°C with gentle stirring until the hexanediol melted. Then 50 μl of dry triethylamine was added to the mixture followed by drop-wise addition of 3.28 g (17.8 mmol) of dry adipoyl chloride. The mixture was gently stirred at 120°C for 48 h until no more HCl was produced. Then, excess (40 g; 100 mmol) of PEG_400_ (carefully dried under reduced pressure) was added at 120°C and the reaction mixture was heated while stirring for another 48 h. The resulting viscous mixture was poured into 50 ml of dry ether and the precipitate was filtered and washed repeatedly with dry ether. The precipitate was then stirred with 100 ml of distilled water and centrifuged at 900 rpm for 4 min. This process was repeated thrice. The resulting polymer was finally dried by overnight freeze drying and characterized by ^1^ H NMR (Bruker Avance III 400 MHz NMR spectrometer; CDCl_3_), IR (infrared) spectroscopy, DSC, SEC and UV–vis spectroscopy. Pol_2000_ with the same fluorescent probe was synthesized and characterized as described previously [4].

### Synthesis of fluorescent Pol_400_ and Pol_2000_ polymers with different end groups

#### A. Conversion to amines

In a dry three-necked 100 ml round-bottom flask fitted with a reflux condenser, 0.03 mmol of Pol_400_ (330 mg) was heated at 120°C until it melted. Then 11.2 mg (0.1 mmol) of chloroacetyl chloride was added slowly under stirring. The mixture was then stirred for 6 h. For conversion to the amine-terminated polymers, the mixture was cooled and 6.8 mg of 25 % (w/w) aqueous ammonia (0.1 mmol of ammonia) was added. The mixture was allowed to stir for another 12 h before workup. The polymer was purified and dried as mentioned before for Pol_400_. Similarly, Pol_2000_ with amine terminal groups was obtained [4].

#### B. Conversion to acid

In a similar experimental set up as mentioned for the conversion to the amines, 10 mg (0.1 mmol) of succinic anhydride was added in portions to 0.03 mmol of molten Pol_400_ polymer and allowed to react for 12 h at 120°C. The purification was performed as described above.

#### C. Estimation of terminal hydroxyl group conversion with trichloroacetyl isocyanate (TAIC)

In an NMR tube with Pol_400_ polymer sample dissolved in CDCl_3_, 10 μl of TAIC was added and the tube was vortexed for 5 min [4]. Then after another 10 min ^1^ H NMR spectra were recorded and the peak appearing at δ = 4.43 ppm was integrated and compared with the value obtained from unmodified polymer to obtain the conversions of the terminal -OH groups [25].

### Synthesis and characterization of nanoparticles

Nanoparticles were prepared by the nanoprecipitation method using a slight modification of the method described by Khoee et al. [71]. First, 10 mg of tri-block copolymer (Pol_400_ or Pol_2000_ to obtain PNP_90_ and PNP_45_, respectively) were dissolved in 2 ml THF with mild heating (~35°C) and the solution was then injected into 10 ml of vigorously stirred ultrapure water in an open beaker [4]. The dispersion was stirred for another 30 min. Then THF was removed under reduced pressure and the aqueous dispersion was filtered through 0.2 μm cellulose filters. Essentially no material remained on the filter. These stock solutions were diluted in measuring flasks to obtain solutions with well-defined concentrations. The hydrodynamic radius of the PNP, both in aqueous dispersions and in F12-K and DMEM cell culture media were determined by DLS at 90^o^ (0.1 μg/ml), and their zeta (ζ) potential (0.1 μg/ml) was obtained with a Malvern Zetasizer. The cell culture mediums (F12-K and DMEM) were also checked by DLS and ζ-potential measurements. While the DLS failed to measure any particulate material > 5 nm in size, the ζ-potential measurements varied between -5 to -10 mV, which can be attributed to the anionic protein molecules originating from the FCS. The serial dilutions of PNP in F12-K or DMEM media were also checked by DLS to exclude any agglomeration of PNP occurring within the tested concentration range. The probability of monomers of the tri-block copolymer leaking out of the PNP resulting in the disintegration of the PNP was excluded by both the SEC and DLS performed at different intervals.

### Scanning Electron Microscopy (SEM)

A clean circular cover glass, 8 mm diameter, (Menzel, Braunschweig, Germany) was fitted on a sample holder by carbon adhesive tabs (EMS, Washington, USA) and 50 μl of an aqueous suspension of PNP was put on the glass and the water was allowed to evaporate until the PNP that remained behind were completely dried [4]. The dry sample was sputter-coated with 2 nm tungsten (MED 020, Leica, Vienna, Austria). Samples were analyzed at 2 kV at room temperature in a field emission scanning electron microscope (Magellan 90, FEI, Eindhoven, the Netherlands).

### Cell lines

Rat alveolar macrophage (NR8383) and human colonic adenocarcinoma (Caco-2) cells were obtained from ATCC (Manassas,VA) [14,15]. The NR8383 and Caco-2 cells were cultured in 150 cm^2^ cell culture flasks with 25 ml F12-K culture medium (Gibco 21127) and DMEM medium, respectively, both supplemented with 10 % (v/v) heat-inactivated fetal calf serum (FCS) and 0.1 % (v/v) gentamicin, in a humidified atmosphere containing 5 % CO_2_ at 37°C.

### Cytotoxicity measurement by MTT assay

#### A. NR8383 cells

An NR8383 cell suspension was centrifuged at 140 *g* for 5 min before re-suspending the cell pellet in F12-K medium followed by counting and adjusting the cellular concentration to 2 × 10^5^ cells/ml. The cells were then seeded in a 96-well plate (50 μl/well) and the plate was kept in a 5 % CO_2_ incubator at 37°C for 24 h. Subsequently, 50 μl of serial dilutions of freshly prepared and well-vortexed different PNP_90_ in F12-K medium were added to the cells to obtain the required final concentrations [14,15]. The concentration range of 0-400 μg/ml was chosen because these concentrations appeared to detect the differences in toxic responses of the cells to the different PNP. This was followed by incubation for another 24 h after which 5 μl of MTT solution in PBS (5 mg/ml) was added to each well and the plate was incubated for another 4 h. Then 100 μl of pure dimethylsulfoxide (DMSO) was added to each well to dissolve the formazan crystals. As the NR8383 cells are a suspension cell line, the medium in the wells of the 96-well plates could not be evacuated before addition of DMSO to the wells as also described before [72]. The absorption of each well was measured at 562 nm in a 96-well plate reader and the background absorption at 612 nm was subtracted. Mitochondrial metabolic activity for each concentration of PNP was expressed as % of the corresponding negative control reading. Medium without PNP and medium with Triton-X (0.1 %) were used as negative and positive controls respectively. Additional control experiments were performed in order to exclude a possible interference with the absorption by the PNP themselves by measuring the absorbance values in a similar set-up after mixing MTT reagent as well as only F12-K medium with different dilutions of PNP_90_.

#### B. Caco-2 cells

The Caco-2 cells were plated at a concentration of 10^5^ cells/ml in a 96-well plate (100 μl/well) and were incubated at 37°C for 24 h [14,15]. Then different freshly prepared and well-vortexed PNP_90_ in DMEM medium were added to the cells (100 μl/well) to achieve the final concentrations followed by further incubation of 24 h at 37°C. 5 μl of MTT solution (in PBS) was then added to each well followed by an incubation of 4 h. Each well was then carefully emptied (because unlike NR8383 the Caco-2 cells attach to the bottom of the wells) without dislodging the precipitated crystals and the crystals were dissolved in pure DMSO (100 μl/well). Finally, each well was measured as mentioned above. Control experiments, as mentioned before, were also done.

#### C. Phagocytic index measurement in NR8383 cells

An NR8383 cell suspension (2 × 10^5^ cells/ml) was seeded in a 96-well plate (50 μl/well) in F12-K medium, followed by addition of 50 μl/well of serial dilutions of freshly prepared and well-vortexed PNP_90_ in F12-K medium to obtain the required final test concentrations of PNP [14,15]. Plain F12-K medium without PNP_90_ and medium containing 100 μM CuSO_4_ were used as negative and positive control, respectively. After 24 h the cells were exposed to yellow-green fluorescent latex beads (1 μm size) at a ratio of beads to cells in each well of 50:1. After 4 h of incubation counting samples were taken from the wells and viewed first under a fluorescent microscope to visualize the fluorescent beads, followed by bright-field view to visualize the cells. Samples were also taken out of each well to assess the cell viability by trypan blue exclusion test. The phagocytic index was determined by calculating the average number of fluorescent beads phagocytosed per viable cell and expressed as % of the negative control. Control experiments were run with only PNP in absence of fluorescent latex beads (1 μm) and no phagocytic vacuole inside the NR8383 cells could be seen.

### Measurement of intracellular ROS by DCFH-DA assay

#### A. NR8383 cells

The cell suspension was adjusted to 2 × 10^5^ cells/ml and seeded in a 96-well plate (50 μl/well) in F12-K medium. 50 μl/well of serial dilutions of freshly prepared and well-vortexed PNP_90_ in F12-K medium were added to obtain the required final test concentrations of PNP. A concentration of 10 mM H_2_O_2_ was used as positive control, and F12-K medium without PNP as negative control. After 6 h of exposure to the PNP, 5 μl of a 20 mM solution of DCFH-DA was added to each well and the plates were incubated for another 18 h in a 5 % CO_2_ atmosphere at 37°C. The fluorescence was then measured on a fluorometer (*λ*_*ex*_ = 485 nm and *λ*_*em*_ = 538 nm). The fluorescence induction factor for each concentration of PNP_90_ was calculated by dividing the reading of each well by the average reading of the negative control and expressed as %. Control experiments were performed by incubating the PNP_90_ at their test concentrations with DCFH-DA in the absence of cells to check the possibility of a positive fluorescence reading caused by reaction of DCFH-DA with PNP_90_ alone [14,15].

#### B. Caco-2 cells

The cells were suspended in DMEM medium to a concentration of 10^5^ cells/ml after trypsinization and were seeded in a 96-well plate (100 μl/well). After 24 h the cells were exposed to 100 μl/well of final concentrations of freshly prepared and well-vortexed different PNP_90_ in DMEM medium. Following another 6 h of PNP_90_ exposure, 5 μl of a 20 mM solution of DCFH-DA was added to each well. The plate was further incubated for 18 h before measurement of the fluorescence was carried out as described above.

### Measurement of mitochondrial membrane potential (ΔΨ_m_)

The NR8383 and Caco-2 cells were plated and exposed to serial dilutions of freshly prepared and well-vortexed PNP of both sizes (45 and 90 nm) as mentioned before. The mitochondrial membrane potential (ΔΨ_m_) was measured by a commercially available kit from Invitrogen (MitoProbe^TM^; Transition Pore Assay Kit; catalogue no. M34153) and expressed as % of negative control (0 μg/ml). A 100 μM solution of ionomycin in DMSO (supplied with the kit) and F12-K or DMEM medium without PNP were used as positive and negative controls, respectively.

### Measurement of intracellular ATP content

The NR8383 and Caco-2 cells were seeded in a 96-well plate and exposed to different freshly prepared and well-vortexed PNP_90_ and PNP_45_ as mentioned before. After 24 h the intracellular ATP content of each well was measured by a commercial ATP measuring kit (Sigma Aldrich, Product No. FLASC) and results were expressed as % of negative control. Cells exposed to medium without PNP and to medium with 75 mM 2,4-DNP (2,4-dinitrophenol) were used as negative and positive controls, respectively.

### Measurement of TNF-α release in NR8383 cells

The NR8383 cells were seeded in a 96-well plate and exposed to different concentrations of freshly prepared and well-vortexed PNP_90_ of each type, as mentioned above. After 24 h the supernatants were collected, centrifuged at 1000 *g* for 10 min, and then spectrophotometrically analyzed for the TNF-α content with a commercial rat TNF-α kit (Invitrogen), using the procedure from the manufacturer’s manual. Medium without PNP and medium with 0.1 μg/ml lipopolysaccharide (LPS) [73] were used as negative and positive controls, respectively.

### Confocal laser scanning microscopy (CLSM)

For performing CLSM a drop of the NR8383 or, Caco-2 cell suspension was placed on a glass slide and viewed through an oil immersion lens (100×) of a confocal microscope (Zeiss Exciter). For assessment of the average fluorescence intensity, readings from 20 individual cells from five different optical fields (for each PNP_90_) in focus selected from five separate experiments (n = 5) were used. All the measurements were done at the same excitation wavelength (*λ*_*ex*_ = 488 nm and *λ*_*em*_ = 543 nm), laser power, pinhole opening and detector gain. To exclude any background fluorescence, control samples of NR8383 or, Caco-2 cells not exposed to the different PNP_90_90, were also investigated by CLSM. These NR8383 or, Caco-2 cells did not show any background fluorescence. Non-fluorescent PNP_90_90 were also tested to exclude any additional fluorescence from the PNP.

### Effect of inhibition of endocytosis

#### A. Inhibition of endocytosis by performing the experiment at 4°C

The NR8383 and Caco-2 cells (after trypsinization) were seeded and exposed to non-toxic 1 μg/ml concentrations of different PNP_90_90 in a 96-well plate as mentioned above and were both pre-incubated and incubated at 4°C. Results of the CLSM images (*λ*_*ex*_ = 488 nm; *λ*_*em*_ = 543 nm) were compared to results from similar incubations performed at 37°C [4]. A figure of colony of NR8383 cells that have taken up fluorescent PNP_90_90 -NH_2_ is provided as Additional file 7. Control experiments with cells exposed to PNP without a fluorescent probe (non-fluorescent PNP) of similar sizes and surface groups were done. Non-fluorescent PNP in absence of cellular system were also tested by CLSM and did not show any fluorescence signal. The results for PNP_90_ were then compared with PNP_45_[4].

#### B. Inhibition of endocytosis by exposure to a mixture of 2-deoxyglucose and sodium azide

An NR8383 or Caco-2 cell suspension was exposed to a mixture of 50 mM 2-deoxyglucose and 10 mM sodium azide [74] for 30 min at 37 ºC before being centrifuged and generously washed with PBS at least three times to remove the exposure medium. Finally, the cells were plated and exposed to different PNP_90_ at 37°C as described before. The results for PNP_90_ were then compared with PNP_45_[4].

### Inhibition of clathrin and caveolin receptor-mediated endocytosis

The NR8383 and Caco-2 cells were exposed to 450 mM sucrose (to inhibit clathrin receptors) [75] or to 1 μM methyl-beta-cyclodextrin (MβCD) (to inhibit caveolin receptors) [44] for 30 min before being washed, plated and exposed to different PNP_90_ at 37°C. Control experiments were done by incubating the cells with 10 μg/ml of Alexa Fluor 488 nm-conjugated transferrin (selective inhibitor for clathrin/*λ*_*ex*_ = 495 nm and *λ*_*em*_ = 519 nm) [76] or 5 μg/ml of Alexa Fluor 488 nm-conjugated cholera toxin subunit-B (selective inhibitor for caveolin/*λ*_*ex*_ = 495 nm and *λ*_*em*_ = 519 nm) [76] for 30 min on ice followed by thorough washing with PBS thrice and then performing CLSM on the cells in order to confirm the blockade of the clathrin and caveolin receptors. It was seen that the inhibitors used (sucrose and MβCD) could block > 90 % of the normal uptake of transferrin or cholera toxin for both the cell lines (Additional file 8) without any additional cytotoxicity. The results for PNP_90_ were then compared with PNP_45_[4].

### Inhibition of mannose receptor mediated endocytosis

The NR8383 cells were exposed to a 2 mg/ml concentration of α-mannan for 2 h in order to inhibit the mannose receptors [77] before being washed, plated and exposed to different PNP_90_ and PNP_45_ at 37°C.

## Competing interests

The authors declare no competing interests and are alone responsible for the content of the article.

## Authors’ contributions

SB performed the synthesis of both different polymer and PNP including their characterizations. SB and KF (supervised by SB) performed the cellular toxicity experiments. SB and DE (supervised by JvdG) performed the CLSM experiments. ATMM, GMA, IMCMR and HZ supervised the entire project. SB, ATMM, GMA, IMCMR and HZ wrote the manuscript. All authors read and approved the final manuscript.

## Supplementary Material

Additional file 1 ^**1**^** H NMR spectrum of PEG**_**400**_**-PHA-PEG**_**400**_** polymer in CDCl**_**3**_** showing no peak(s) at δ = 4.43 ppm.** Click here for file

Additional file 2 **SEC trace of PEG**_**400**_**-PHA-PEG**_**400**_** polymer in THF.** Click here for file

Additional file 3 **IR spectrum of Pol**_**400**_** [PEG**_**400**_**-PHA-PEG**_**400**_**] in carbon tetrachloride (CCl**_**4**_**).** Click here for file

Additional file 4 ^**1**^** H NMR spectrum of unmodified PEG**_**400**_**-PHA-PEG**_**400**_**polymer to which TAIC is added.** The presence of a trifurcated peak at δ = 4.43 ppm results from the reaction of the free terminal hydroxyl groups with TAIC. Click here for file

Additional file 5 **SEM pictures of PNP**_**90**_**-NH**_**2**_**, PNP**_**90**_**-OH and PNP**_**90**_**-COOH. Scale bars show 100 nm.** Click here for file

Additional file 6 ***z*-stack imaging in NR8383 cells after 24 h exposure to the PNP**_**90**_**-OH at 4 **^**o**^**C showing that the PNP were actually inside the cells and not bound to the cell membrane (*λ***_***ex***_** = 488 nm;*λ***_***em***_** = 543 nm).** Slide 1 showed the bottom and slide 12 showed the top sections of the cell with thickness of each slice ~400 nm. Click here for file

Additional file 7 **CLSM picture of some NR8383 cells that have taken up fluorescent PNP**_**90**_**90-NH**_**2**_**.** Click here for file

Additional file 8 **Uptake of green fluorescent transferrin and cholera toxin subunit-B by NR8383 and Caco-2 cells.** Control: no blocking of the receptors. Test: Upon selectively blocking the clathrin receptors by hypertonic sucrose or caveolin receptors by MβCD. Click here for file
