# Supplementary figures and images for: Cytotoxicity and cellular uptake of tri-block copolymer nanoparticles with different size and surface characteristics
Source: Part Fibre Toxicol. 2012 Apr 30;9:11. doi: 10.1186/1743-8977-9-11 (PMC3419642; doi:10.1186/1743-8977-9-11)

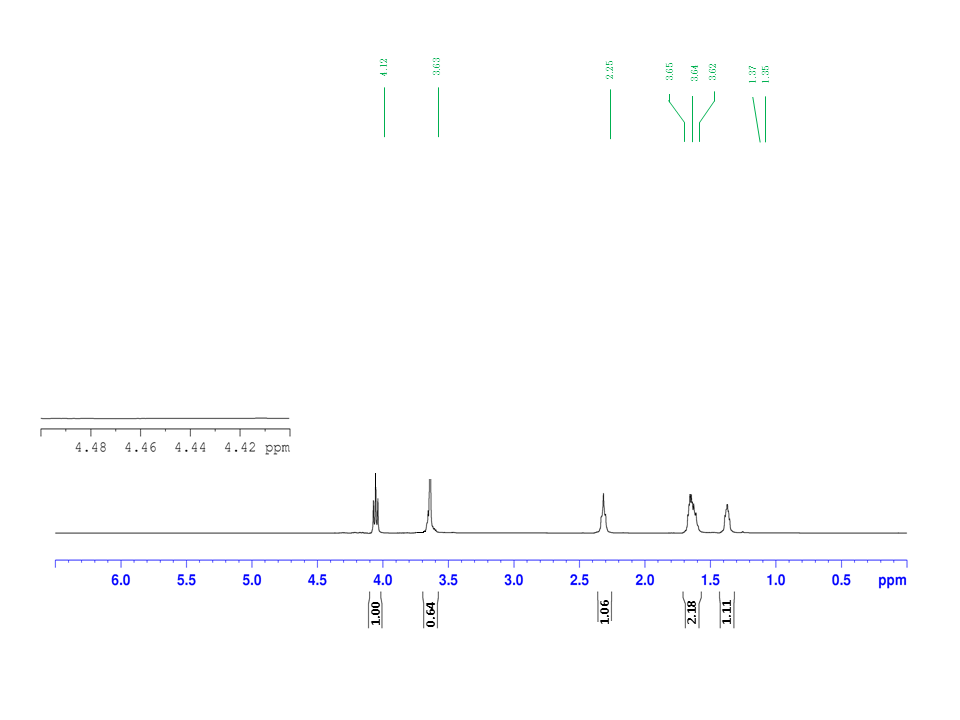

Supplement: Additional file 1 — 1 H NMR spectrum of PEG400-PHA-PEG400 polymer in CDCl3 showing no peak(s) at δ = 4.43 ppm. [file 1743-8977-9-11-S1.tiff]

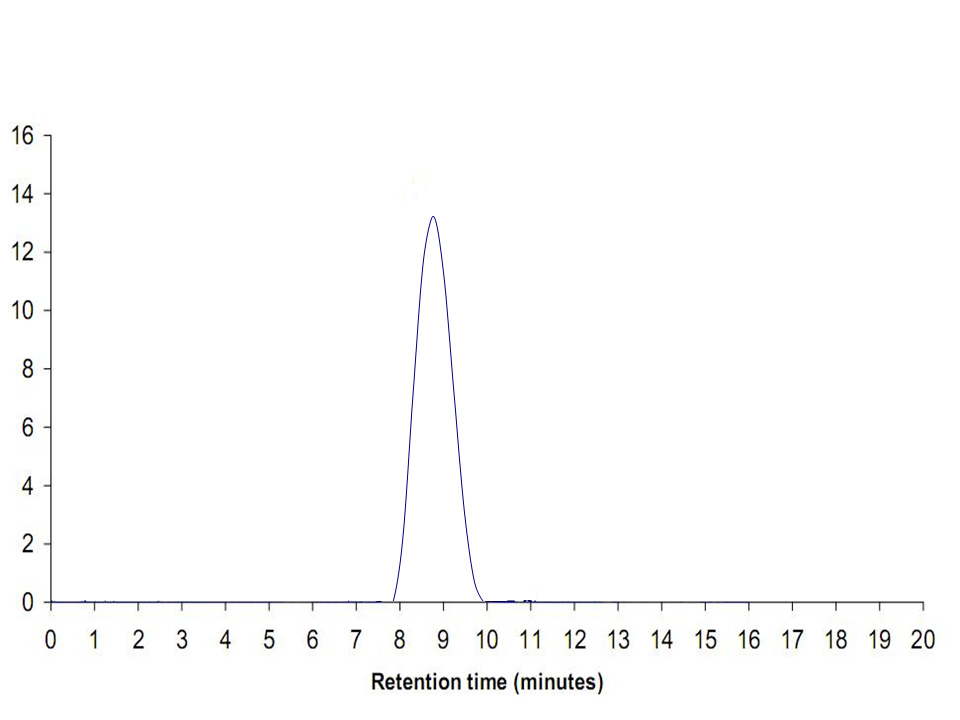

Supplement: Additional file 2 — SEC trace of PEG400-PHA-PEG400 polymer in THF. [file 1743-8977-9-11-S2.tiff]

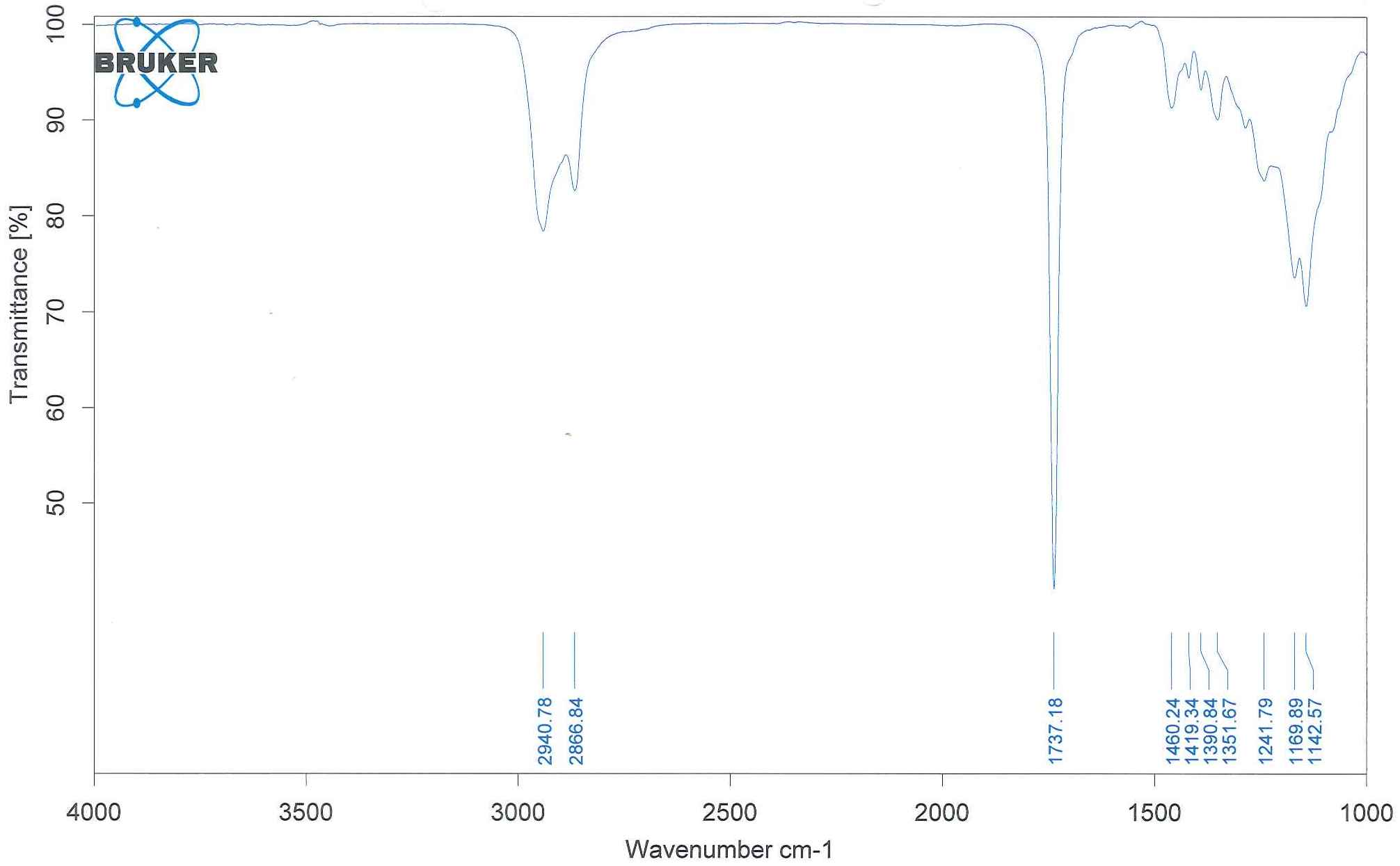

Supplement: Additional file 3 — IR spectrum of Pol400 [PEG400-PHA-PEG400] in carbon tetrachloride (CCl4). [file 1743-8977-9-11-S3.tiff]

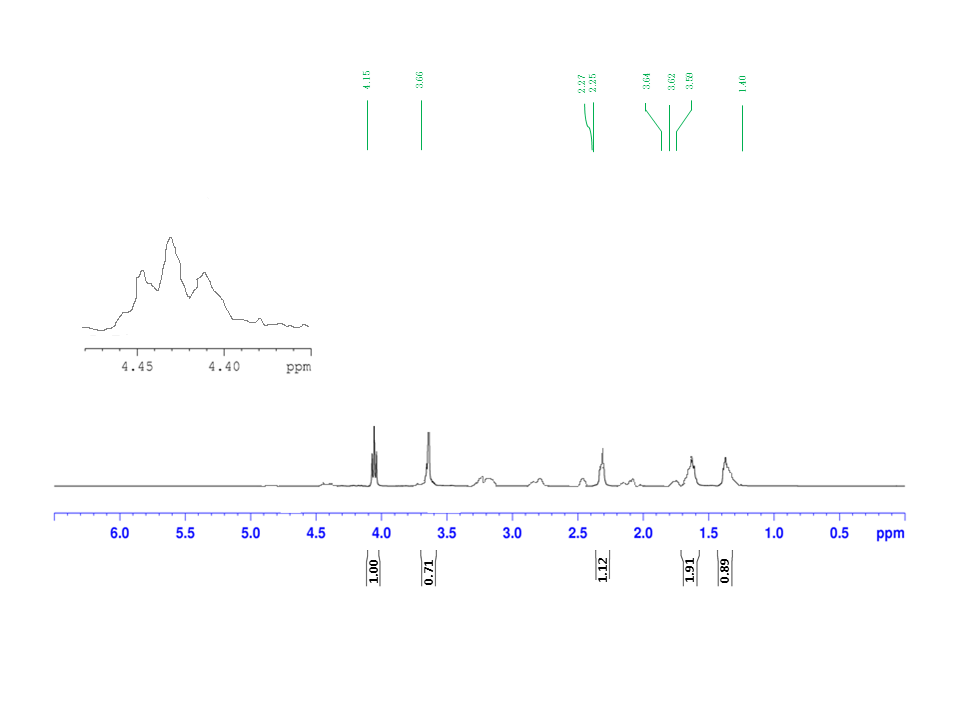

Supplement: Additional file 4 — 1 H NMR spectrum of unmodified PEG400-PHA-PEG400polymer to which TAIC is added. The presence of a trifurcated peak at δ = 4.43 ppm results from the reaction of the free terminal hydroxyl groups with TAIC. [file 1743-8977-9-11-S4.tiff]

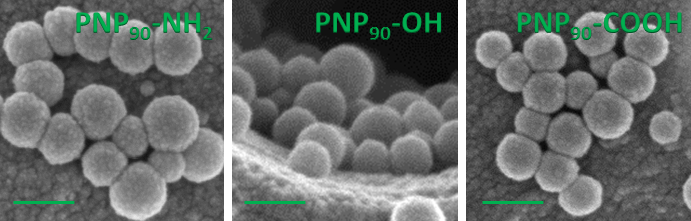

Supplement: Additional file 5 — SEM pictures of PNP90-NH2, PNP90-OH and PNP90-COOH. Scale bars show 100 nm. [file 1743-8977-9-11-S5.tiff]

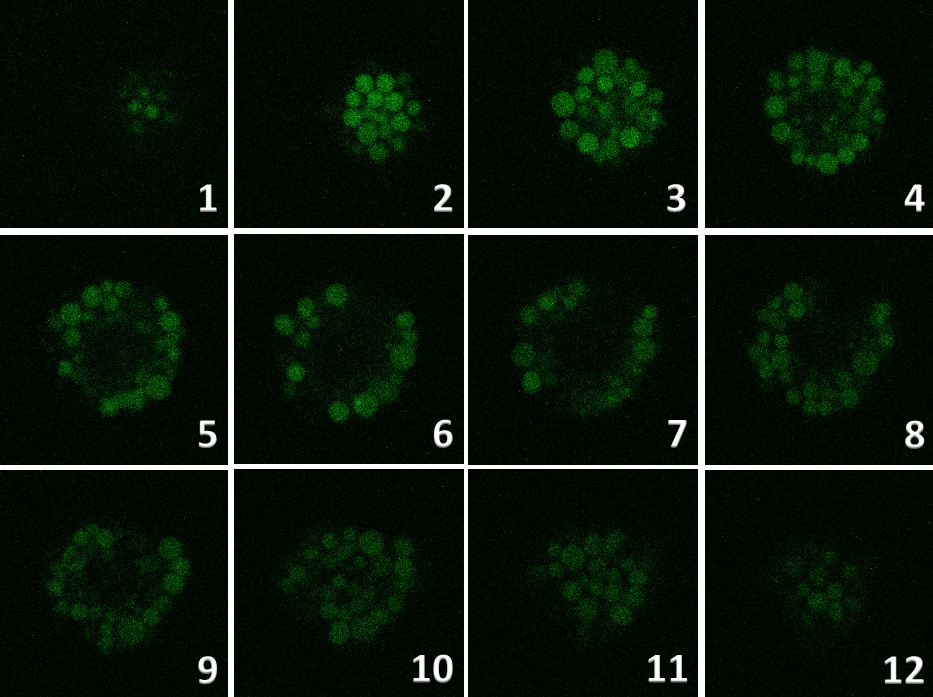

Supplement: Additional file 6 — z-stack imaging in NR8383 cells after 24 h exposure to the PNP90-OH at 4 oC showing that the PNP were actually inside the cells and not bound to the cell membrane (λex = 488 nm;λem = 543 nm). Slide 1 showed the bottom and slide 12 showed the top sections of the cell with thickness of each slice ~400 nm. [file 1743-8977-9-11-S6.tiff]

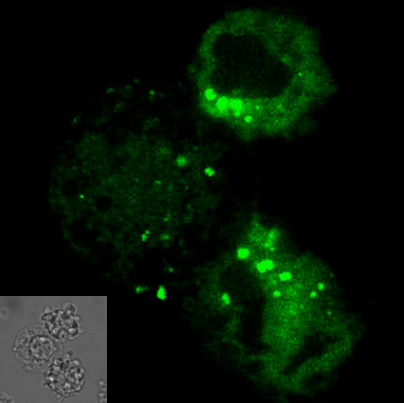

Supplement: Additional file 7 — CLSM picture of some NR8383 cells that have taken up fluorescent PNP9090-NH2. [file 1743-8977-9-11-S7.tiff]

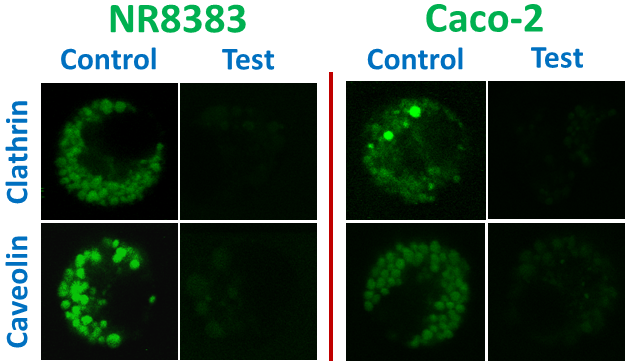

Supplement: Additional file 8 — Uptake of green fluorescent transferrin and cholera toxin subunit-B by NR8383 and Caco-2 cells. Control: no blocking of the receptors. Test: Upon selectively blocking the clathrin receptors by hypertonic sucrose or caveolin receptors by MβCD. [file 1743-8977-9-11-S8.tiff]
